# The Registration and Inspection of Children's Homes

**Published:** 1902-03-22

**Authors:** 


### The Institutional Workshop.

### THE REGISTRATION AND INSPECTION OF CHILDREN'S HOMES.

By a Correspondent.

The conference which took place on March 6th at the offices of the Church of England Waifs and Strays Society in Savoy Street marks an important advance in the handling of this difficult question. General resolutions were unanimously passed in favour of the registration and inspection of homes for children supported by funds obtained from the charitable public, and the representative character of the gathering goes far to destroy the argument of those who would represent the movement in favour of registration as aiming at curtailing the wholesome liberties of individual charities. It is not outside bodies petitioning to place these homes under supervision, though outside bodies such as the Charity Organisation Society and that for the Protection of Children from Cruelty have much to say in support of the measure. It is the homes themselves, the founders and managers of some of the largest organisations for aiding destitute children, who are petitioning the State to grant them registration and inspection.

It is one of the curious anomalies of our social system that the evils which it is proposed to attack by registration can only be assailed indirectly.

It is notorious that many homes are "run" by greedy speculators who take in orphan children and make enormous profits from the sums which they induce the public to subscribe by means of specious appeals. The children thus housed often suffer in mind, body, and morals to an extent which comes a good deal short of the actual cruelty inviting criminal action. The people who subscribe for their benefit also suffer, inasmuch as the money they give to help orphan children is misapplied. The hardships inflicted on the orphans who are exploited for the benefit of imposters is incomparably greater than the misappropriation of certain small sums of money heedlessly given. Yet it is on the latter side alone that the arm of the law can be enlisted with any hope of success.

In other words, it may be impossible to close unsatisfactory homes even although the clearest evidence be given of their deficiencies; but it may be possible to prohibit their promoters from appealing for public support.

We believe that a measure such as that now proposed would put an immediate stop to the disgraceful traffic in orphan children now carried on under the very eyes of powerless authorities. It is not to be supposed that it would at once annihilate the professional philanthropist; but it would at least turn his attention to other and less pitiably helpless prey.

But it would do far more than this. Some of those intimately connected with the management of important homes spoke at the conference with practical experience of the assistance which trained inspection would give to the men and women on whom the burden of the work rests. The permanent officials of these homes have no time to spare for visiting other homes, comparing systems and obtaining new lights on the recurring problems of the work they have most at heart. An inspector conversant with all systems could suggest and enlighten even where he did not authoritatively recommend, and we believe his advice would be often most anxiously sought on many difficult points.

In cases, moreover, where the inspector felt it to be his duty to make strong recommendations, the position of the charity in appealing to their subscribers for the necessary improvements would be definitely strengthened. Even the most generous donors are apt to turn restive if they are appealed to for things which they consider "fads"—fire escapes, new drains, or bathrooms. But if the Government inspector throws out a hint that these things must be set in hand if he is to pass the buildings at his next visit, the subscribers may grumble but they will find the money.

We confess, however, that in our opinion the more important homes for children, those which are at this moment appealing for registration, are not those which will benefit most from the measure. And many, too, of the smallest orphanages are at the present moment admirably managed, and could gain little from inspection.

There is a large class of homes which lie on the borderland between good and evil. Founded with the best intentions, they are carried on with a sincere wish to benefit the children received, but the difference between desire and fulfilment is lamentably apparent to those who come to an intimate knowledge of these households.

To bring children together from different homes and provide them with all things necessary for their well-being, present and future, is a work demanding special knowledge and experience. The amateur, however full of zeal for his neighbours' children, probably blunders at every step. He blunders at the very beginning in his selection, mixing the children of respectable parentage with those of vicious antecedents, the feeble-minded with the vigorous. He proceeds on the assumption that the children need have no higher standard of accommodation than what they would have had in their own homes, forgetting that family life is a natural state and that nature provides compensations, but that artificial conditions such as prevail in the life of the institution must be governed by strict laws of hygiene at the risk of engendering the gravest evils. The amateur, again, is often extravagant where he should be economical and terribly parsimonious where he ought to be freehanded; and, lastly, he very rarely indeed understands how to choose the right person for matron, secretary, or whoever is in direct charge of the home. And an incalculable amount of harm results from the wrong people being foisted into these responsible positions.

Inspection would in the course of a few years gradually and almost imperceptibly raise the standard in homes such as we have been alluding to, and would introduce happier methods in all the countless instances where not zeal but merely knowledge is lacking.

If inspection is to prove a real support and assistance to the numerous small homes for girls which are now doing good if sometimes imperfect service to the State, the inspectors for these homes should undoubtedly be women. The faults which are met with in these little establishments are such as women can most easily detect. Defects in washing and sanitary accommodation; deficiency in clothing, especially underclothing; degrading punishments, and the retaining of refractory girls after it has become evident that they are gaining no good and injuring the general discipline; such are some of the evils which may have to be rectified. The advent of a male inspector into some of these little domestic circles where the accounts are rudimentary and the arrangements a series of makeshifts, would bring well-founded consternation. But a homely, sensible woman inspector, ready to enter into difficulties and suggest expedients, need not be dreaded by the most humble-minded matron, and ought, after one or two visits, to ensure her own welcome.

There is a deep-rooted objection by the public to any transfer to the State of the voluntary charitable work which is the great pride of Englishmen. But for this very reason the petition of the charities themselves for protection against those who prey upon them should meet a ready acceptance.

